# Expression and Secretion of CXCL-8 and CXCL-10 From *Mycobacterium Bovis* BCG-Infected Human Epithelial Cells: Role of IL-4

**DOI:** 10.1155/MI/2006/67451

**Published:** 2006-02-14

**Authors:** Patricia Méndez-Samperio, Elena Miranda, Abraham Vázquez

**Affiliations:** Departamento de Inmunología, Escuela Nacional de Ciencias Biológicas, IPN. Carpio y Plan de Ayala, México, D.F. 11340, México

## Abstract

CXC chemokine release can be modulated by Th2-derived cytokines. Interleukin(IL)-4 is one of the cytokines that are the hallmark of the Th-2 response, and plays an important role in human tuberculosis. In the current study, we investigated the effect of IL-4 on chemokine production by human epithelial cells infected with *Mycobacterium bovis* bacillus calmette-guérin (BCG). Gene expression of CXCL-8 and CXCL-10 was determined by the reverse transcription (RT)-polymerase chain reaction method. The levels of immunoreactive CXCL-8 and CXCL-10 were determined by enzyme-linked immunosorbent assay. We found that, although *M. bovis* BCG induced gene expression of CXCL-8 and CXCL-10 in *M. bovis* BCG-infected human epithelial cells, CXCL-8 mRNA level was significantly reduced by IL-4, whereas no significant effect of IL-4 was observed on CXCL10 mRNA level. In addition, IL-4 decreased CXCL-8 (in a graded and significant manner) but not CXCL-10 secretion. These results were further confirmed, since a significant reversion was obtained with a neutralizing antibody to human IL-4, whereas an isotype-matched control antibody had no significant effect on CXCL-8 secretion. Furthermore, we found a similar effect of IL-4 on *M. bovis* BCG-induced CXCL-8 and CXCL-10 secretion by using other human epithelial A549 cell line. Collectively, these data demonstrate that *M. bovis* BCG-infected human epithelial cells can have an active role in a local inflammatory immune response via the secretion of CXC chemokines which can be selectively regulated by Th2-derived cytokines.

## INTRODUCTION

The hallmark of *Mycobacterium tuberculosis* infection is characterized by an inflammatory response culminating in the formation of a granuloma [1]. Chemokines play a key role in controlling the migration of cell types found within the lung during *M. tuberculosis* infection [2]. Chemokines are divided into four subgroups on the basis of structural criteria with the majority classes as CC or CXC chemokines [3]. CXC chemokines include CXCL-8 (interleukin-8), which is a potent neutrophil, monocyte, and T-lymphocyte chemoattractant [4, 5], and CXCL-10 (interferon-inducible protein-10), which attracts T-lymphocytes [6] but not neutrophils. These CXC chemokines are produced by inflammatory cells such as monocytes/macrophages [7, 8] and by human epithelial cells [9, 10] after exposure to *M. tuberculosis* and *M. bovis*. Release of chemokines by human epithelial cells, which are considered the major cellular source of chemokines in the lung [11], can be modulated by Th2-derived cytokines [12]. Recent data have indicated increased production of interleukin(IL)-4, a Th2 cytokine, by cells from patients with tuberculosis [13]. In addition, active tuberculosis has been associated with increased Th2 activity in vivo [14]. This is in agreement with the observation that patients with tuberculosis have raised levels of IgE antibody [15]. Although the Th2 cytokines have been demonstrated to downregulate chemokine secretion in a number of cellular models [12, 16, 17], little is known about their effects on chemokine release in *Mycobacterium bovis* bacillus calmette-guérin- (BCG-) infected human epithelial cells. Since the *M. bovis* BCG strain is the only vaccine currently available for protection against tuberculosis [18], this study aimed to investigate the effect of IL-4 on *M. bovis* BCG-induced CXCL-8 and CXCL-10 secretion and gene expression in human epithelial cells.

## MATERIALS AND METHODS

### Reagents

Recombinant human IL-4, antihuman IL-4, and isotypematched control antibody were purchased from Santa Cruz Biotechnology, Inc (Santa Cruz, Calif, USA)

### Mycobacterial cultures

*Mycobacterium bovis* (ATCC 35733) was obtained from the American Type Culture Collection (Rockville, Md, USA). *M. bovis* was grown at 37°C in Sauton medium for 2 weeks. Cultures were centrifuged at 800 rpm for 10 min and then washed three times in medium. Mycobacteria were resuspended in medium and aliquots of the stock were kept at −70°C.

### Epithelial cell culture

The human epithelial HEp-2 cell line was originally acquired from the American Type Culture Collection (Rockville, Md, USA). Cells were cultured in minimum essential medium Eagle supplemented with 2 mM of L-glutamine, 1 mM of sodium pyruvate, 0.1 mM of nonessential amino acids, and Earle's BSS adjusted to contain 1.5 g/l of sodium bicarbonate and 10% heat-inactivated foetal bovine serum (Gibco-BRL, Rockville, Md, USA). Cells were grown at 37°C in a humidified 5% CO_2_ atmosphere. Cells were used at approximately 80%–90% confluence before performing experiments. The human alveolar epithelial A549 cell line was maintained in a humidified 5% CO_2_ atmosphere in Dulbecco's modified Eagle's medium.

### Treatment of epithelial cells with IL-4

Cells (10^5^/well) were preincubated in growth media or different concentrations of IL-4 for 2 h before infection with *M. bovis* using an opsonized bacteria-to-cell ratio of 5 : 1. IL-4 did not affect cell viability at the concentrations used. Neutralizing antibody to IL-4 was added to some cultures. Unstimulated cells, cultured in media alone, served as a negative control. The culture medium for the detection of chemokine release was harvested for analysis after 24 h.

### Assessment of CXCL-8 and CXCL-10 mRNA expression by RT-PCR

mRNA levels were assessed using reverse transcription- (RT-) PCR assay. In these experiments, HEp-2 cells were treated with IL-4 (50 ng/mL) for 2 h, and were then infected with *M. bovis* BCG at an MOI = 5. Total RNA from cells was obtained using TRIzol reagent (Life Technologies, Rockville, Md, USA) as per the manufacturer's instructions. Cellular RNA (1 μg) was reverse transcribed to cDNA. The mRNA of the chemokines CXCL-8 and CXCL-10 were analyzed by PCR essentially as described [19]. To verify that equal amounts of undegraded RNA was added in each RT-PCR reaction, GAPDH was used as an internal standard. Amplified PCR products were detected using 2% agarose ethidium bromide gel electrophoresis and photographed.

### Quantification of CXCL-8 and CXCL-10 by ELISA

The levels of immunoreactive CXCL-8 and CXCL-10 were determined with cytokine-specific commercial ELISA kits as per the manufacturer's instructions (R&D Systems, Minneapolis, Minn, USA). The lower limit of sensitivity of the CXCL-8 assay was 3 pg/mL, and that of the CXCL-10 assay was 11 pg/mL.

### Statistics

Data are presented as means from at least three separate experiments with standard errors of the means (SEMs). Statistical significance of differences was assessed by Student's *t*-test. The value of *P* ≤ .05 is taken as statistically significant.

## RESULTS

### Effect of IL-4 on M. bovis BCG-induced CXCL-8 and CXCL-10 gene expression

**Figure 1:**
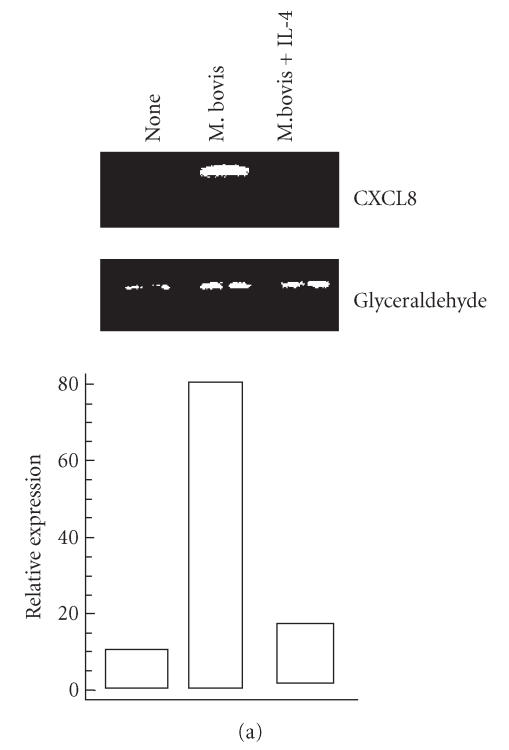

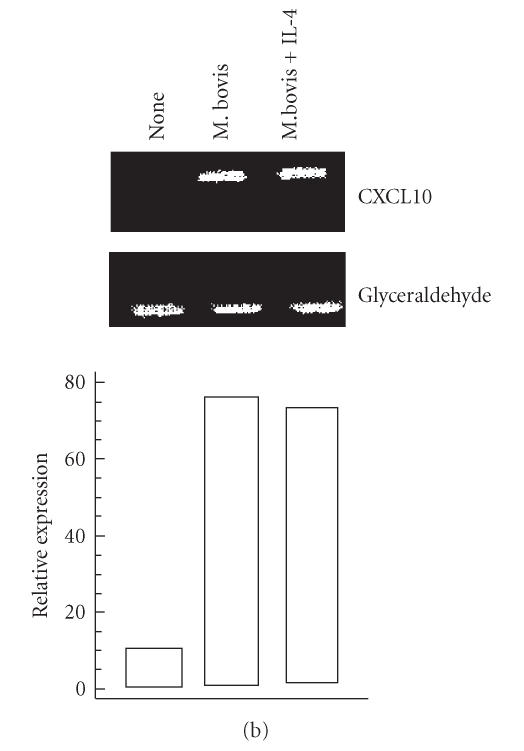
IL-4 has divergent effects on *M. bovis* BCG-induced chemokine gene expression. HEp-2 cells were treated with IL-4 (50 ng/mL) prior to infection with *M. bovis* BCG. After incubation total RNA was isolated and the levels of (a) CXCL-8 mRNA or (b) CXCL-10 mRNA were measured by RT-PCR method. PCR products were run on a 2% agarose gel containing ethidium bromide. The results depicted are representative of three independent experiments. GAPDH, glyceraldehhyde 3-phosphato dehydrogene probe was used to confirm equal RNA loading. The histograms represent relative transcription rates, which were calculated after normalization to the respective GAPDH signal.

We first investigated the effect of IL-4 on CXCL-8 and CXCL-10 gene expression from human epithelial cells infected with *M. bovis* BCG. To test this, HEp-2 cells were pretreated with or without IL-4 and then infected with *M. bovis* BCG. After incubation total RNA was isolated and the levels of CXCL-8 mRNA or CXCL-10 mRNA were measured by RT-PCR method. As shown in Figures 1(a) and 1(b), CXCL-8 mRNA and CXCL-10 mRNA were expressed after infection with *M. bovis* BCG, and IL-4 reduced gene expression of CXCL-8 in *M. bovis* BCG-infected HEp-2 cells (Figure 1(a)) but not CXCL-10 gene expression (Figure 1(b)). These results indicate that inhibition of *M. bovis* BCG-induced expression of CXCL-8 by IL-4 is a result of transcriptional downregulation of CXCL-8.

### Downregulation of CXCL-8 secretion from human epithelial cells infected with *M. bovis* BCG by IL-4

**Figure 2:**
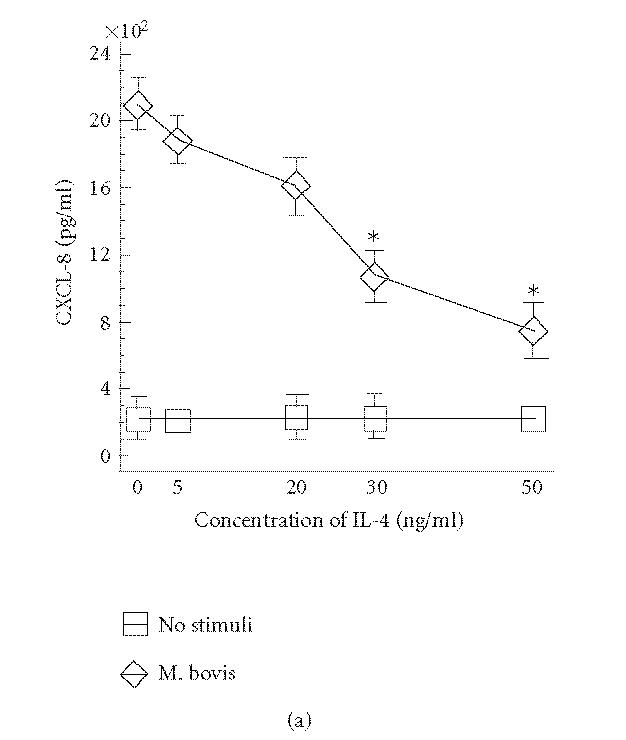

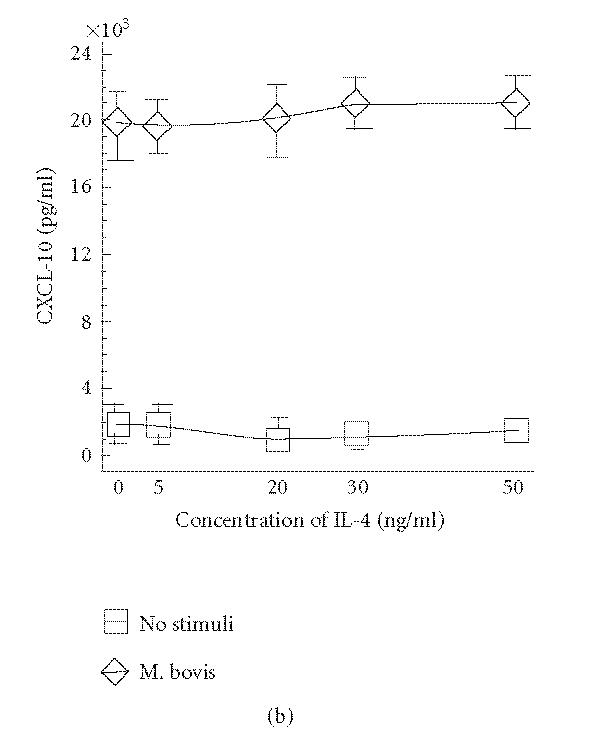
Effect of recombinant IL-4 on *M. bovis* BCG-induced CXCL-8 and CXCL-10 secretion in HEp-2 cells. HEp-2 cells were cultured without a stimulus (no stimuli) or infected by *M. bovis* BCG (5 : 1 bacteria/cell) after 2 h pretreatment with IL-4 (1–50 ng/mL). The (a) CXCL-8 and (b) CXCL-10 protein levels from cellular supernatants were measured by ELISA. Data are presented as mean ± SEM of five independent experiments. Reduction of *M. bovis* BCG-induced CXCL-8 secretion after addition of 30 or 50 ng IL-4 is statistically significant (*P < .01).

**Figure 3:**
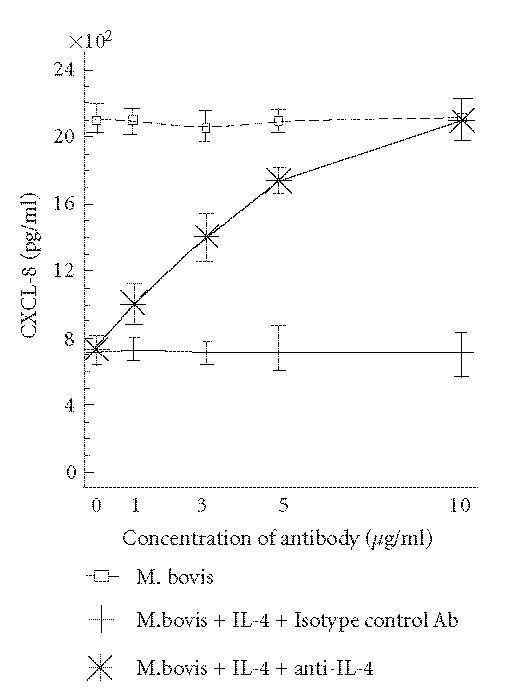
Neutralizing anti-IL-4 antibody significantly reverses the inhibitory effect of IL-4 on *M. bovis* BCG-induced CXCL-8 secretion. HEp-2 cells were pretreated with IL-4 (50 ng/mL) in the presence of different concentrations of anti-IL-4 or an isotype control antibody for 2 h prior to *M. bovis* infection for an additional 24 h at 37°C. CXCL-8 levels were measured by ELISA. The results are the means ± SEM for four separate experiments.

**Figure 4:**
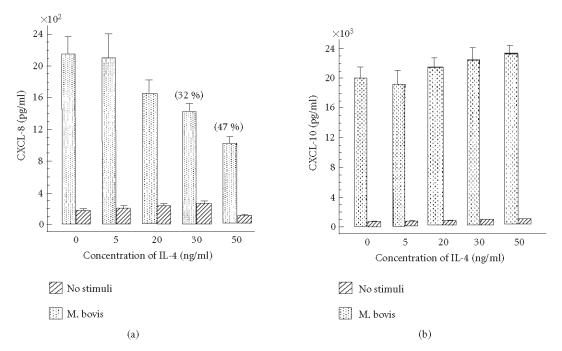
Effect of recombinant IL-4 on CXC chemokine secretion by human alveolar epithelial A549 cells infected with *M. bovis* BCG. Cells were treated with medium (no stimuli) or increasing doses of IL-4 for 2 h prior to infection with *M. bovis* BCG ( MOI = 5). After 24 h incubation, supernatants was collected and (a) CXCL-8 or (b) CXCL-10 were measured by ELISA. Data shown are the mean ± SD of four independent experiments. The percentage in parentheses indicates inhibition in the presence of IL-4 compared with *M. bovis* BCG cultures which did not receive IL-4.

Recent work in our laboratory has demonstrated that human cells secrete CXCL-8 and CXCL-10 in response to *M. bovis* BCG [19]. In this study, we examined the effect of IL-4 at various concentrations on production of CXCL-8 and CXCL-10. HEp-2 cells were pretreated with increasing concentrations of IL-4 and infected with *M. bovis* at MOI = 5. The data in Figure 2(a) demonstrate that IL-4 significantly suppressed *M. bovis* BCG-induced CXCL-8 secretion in a concentrationdependent manner. Maximal inhibition in this series of experiments was 62% when 2073 ± 296 pg/mL of CXCL-8 production with *M. bovis* BCG was reduced to 788 ± 128 pg/mL by IL-4 at 50 ng/mL. In contrast, IL-4 at 50 ng/mL enhanced *M. bovis* BCG-induced CXCL-10 production to a small degree (Figure 2(b)). To further evaluate the specificity of the effect of IL-4 on *M. bovis* BCG-induced CXCL-8 secretion, different concentrations of a neutralizing antibody to IL-4 or an isotype-matched control antibody were added to HEp-2 cells treated with IL-4 (50 ng/mL). As indicated in Figure 3, inhibition of *M. bovis* BCG-induced CXCL-8 secretion by IL-4 was significantly suppressed with 10 μg of anti-IL-4 antibody/mL. It is important to note that an isotype-matched control antibody did not affect the effect of IL-4 on *M. bovis* BCG-induced CXCL-8 secretion (Figure 3). Next, to confirm the regulatory effect of IL-4 on *M. bovis* BCG-induced CXC chemokine secretion, we also used the human alveolar epithelial A549 cell line. As shown in Figure 4, a similar effect of IL-4 on *M. bovis* BCG-induced CXCL-8 and CXCL-10 secretion was observed by using other epithelial A549 cell line. Regulation that correlated with the effect of IL-4 observed for the CXCL-8 and CXCL-10 mRNA gene expression.

## DISCUSSION

Inflammation is a series of coordinated events that depend on leukocyte recruitment to the site of inflammation, in which chemokines play an important role [20, 21]. Our present data demonstrate that the effect of IL-4 on CXCL-8 and CXCL-10 secretion in *M. bovis* BCG-infected human epithelial cells is entirely distinct, since IL-4 decreased *M. bovis*-induced CXCL-8 production but not CXCL-10 secretion. Mycobacterial-induced production of CXCL-8 has been previously demonstrated in human alveolar macrophages, in monocytic THP1 cells, and in bronchoalveolar lavage of pleural fluid from pulmonary tuberculosis patients [7–10, 22]. However, the downregulation of CXCL-8 by IL-4 following *M. bovis* infection is a novel finding. Moreover, further experiments were conducted to determine that the mechanism by which this Th2-derived cytokine downregulates CXCL-8 secretion is mediated at the transcriptional level. According to previous study [23], it has been demonstrated that IL-4 had no effect on TNF-α/IFN-γ-induced CXCL-8 expression and secretion by epithelial cells. Such discordance suggests that the mechanism by which IL-4 regulates CXCL-8 secretion is stimulus-specific. In addition, our results, indicating an important role for transcriptional activation in epithelial cells in response to *M. bovis* BCG, are in agreement with published data which have demonstrated that the induction of chemokine secretion from epithelial cells by mycobacteria occurs at the transcriptional level [24]. On the other hand, the observation that the production of CXCL-10 was not affected by the addition of IL-4 could be ascribed to the activation of the NF-κB pathway induced by the direct interaction of *M. bovis* with the human epithelial cells, since it has been demonstrated that the infection of cells by mycobacteria stimulated a rapid binding of NF-κB to the κB site within the CXCL-10 gene promoter [25]. The effect of IL-4 on transcription factor activation and the CXCL-10 promoter binding as well as on mRNA stability requires further investigation.

Our results are in agreement with the hypothesis that progressive tuberculosis disease might not be due to absence of Th1 response, but rather to the effect of an unusual Th2 response [14]. Recently, it has been demonstrated that progressive tuberculosis disease might be due to preexisting Th2-like activity by inducing toxicity of tumor necrosis factor-α and/or impair bactericidal function [26], we for the first time have demonstrated the selectively effect of IL-4 on chemokine release in *M. bovis* BCG-infected epithelial cells, indicating a novel mechanism of the association of type 2 cytokines with mycobacterial infection. Downregulation of CXCL-8 secretion by IL-4 is likely to be important during the human immune response to *M. bovis* infection, since it provides an opposing Th2-cell mechanism involved in protective host immunity and neutrophil and T-lymphocyte migration can be affected by the reduction in CXCL-8, thus reducing the cell-mediated response.

Of the Th2 cytokines, previous studies have demonstrated that IL-13, which like IL-4, is increased approximately 100-fold compared to controls matched for age and gender [27]. In view of the importance of IL-13, or of the shared receptor between IL-4 and IL-13, our data do not allow us to exclude the possibility that IL-4 synergizes with IL-13 to downregulate CXCL-8 production during infection with *M. bovis* BCG. We are currently investigating this possibility.

In summary, the data from this study demonstrate that in *M. bovis* BCG-infected human epithelial cells IL-4 plays an important role in downregulating CXCL-8 release. Further experimental work is needed to know whether the effect of IL-4 on *M. bovis* BCG-induced CXCL-8 secretion may represent a significant regulatory mechanism in vivo. However, these data may represent an important regulatory mechanism during the immune response to M. bovis BCG, since CXCL-8 induction during mycobacterial infection is a major neutrophil-activating factor and chemotactic.
